# Extraction of Clinically Relevant Temporal Gait Parameters from IMU Sensors Mimicking the Use of Smartphones

**DOI:** 10.3390/s25144470

**Published:** 2025-07-18

**Authors:** Aske G. Larsen, Line Ø. Sadolin, Trine R. Thomsen, Anderson S. Oliveira

**Affiliations:** 1 Department of Chemistry and Bioscience, Aalborg University, DK-9220 Aalborg Oest, Denmark; 2 Department of Materials and Production, Aalborg University, DK-9220 Aalborg Oest, Denmark

**Keywords:** remote monitoring, digital health, machine learning, gait analysis, smartphone, IMU

## Abstract

**Highlights:**

**What are the main findings?**

- Single IMU + CNN-LSTM predicts stride time with <5% errors across hand, pocket, and jacket placements.
- Stance/swing times show moderate errors; double support > 20%, yet all correlate moderately strongly with lab data.

**What is the implication of the main finding?**

- Smartphone-based IMU enables remote, real-world gait tracking.
- Robust predictions across positions and speeds support scalable monitoring of gait disorders.

**Abstract:**

As populations age and workforces decline, the need for accessible health assessment methods grows. The merging of accessible and affordable sensors such as inertial measurement units (IMUs) and advanced machine learning techniques now enables gait assessment beyond traditional laboratory settings. A total of 52 participants walked at three speeds while carrying a smartphone-sized IMU in natural positions (hand, trouser pocket, or jacket pocket). A previously trained Convolutional Neural Network and Long Short-Term Memory (CNN-LSTM)-based machine learning model predicted gait events, which were then used to calculate stride time, stance time, swing time, and double support time. Stride time predictions were highly accurate (<5% error), while stance and swing times exhibited moderate variability and double support time showed the highest errors (>20%). Despite these variations, moderate-to-strong correlations between the predicted and experimental spatiotemporal gait parameters suggest the feasibility of IMU-based gait tracking in real-world settings. These associations preserved inter-subject patterns that are relevant for detecting gait disorders. Our study demonstrated the feasibility of extracting clinically relevant gait parameters using IMU data mimicking smartphone use, especially parameters with longer durations such as stride time. Robustness across sensor locations and walking speeds supports deep learning on single-IMU data as a viable tool for remote gait monitoring.

## 1. Introduction

Gait analysis has traditionally been conducted in laboratory settings. However, advances in sensor minimization, such as inertial measurement units (IMUs), have created new opportunities. These affordable technologies now allow gait analysis in real-world contexts. Previous studies have demonstrated that it is feasible to extract spatiotemporal gait parameters using IMUs firmly fixed to the user. However, the methodological approach and accuracy vary significantly depending on sensor placement and study protocols [1,2,3,4,5,6,7,8,9]. Studies performing gait analysis by fixing IMUs to the user’s pelvis [1,7] and shanks [2] have shown promising results, with no systematic time error. Moreover, a comparison of gait parameter predictions from ten IMU locations demonstrated that locations where people carry smartphones (e.g., front trouser pocket or wrist) yield greater errors compared with fixing the IMU on the foot or pelvis [3]. However, fixing the IMU at the pelvis can be difficult for some people, and fixation in the shanks might not be practical due to clothing choices (e.g., use or not of trousers, the use of tall boots). Therefore, IMU fixation is an overlooked issue for the real-world implementation of remote monitoring methods.

Gait studies simulating smartphone use by placing the IMU in the front trouser pocket have reported moderate to strong intraclass correlation coefficients (ICC range: 0.72–0.95) between experimental and predicted swing times. However, these predictions often showed systematic bias [4,5,6,8]. Remote gait analysis is an emerging area of study, and several companies are beginning to offer commercial solutions. For example, OneStep (OneStep, Tel Aviv, Israel) claims to assess gait quality using smartphone IMU data [4,5,6,7,8], but they require timed walks and smartphone placement in the front trouser pocket. In contrast, Apple Health app (Apple Inc., Cupertino, CA, USA) claims to assess walking steadiness in freely moving users without device constraints. However, the feature is not open source; therefore, alternative solutions are needed for technologies to reach a wider user group.

Recent research demonstrated the feasibility of detecting heel strikes and toe-off events using a surrogate smartphone containing an IMU that was carried by the participant in the hand, trouser pockets, and jacket pockets [10]. Therefore, strict sensor fixation may not be necessary for extracting gait parameters in real-world conditions. This flexibility could improve user adherence and enhance practical applicability. Therefore, the aim of this study was to determine the accuracy of gait parameters (e.g., double support time, stride time, stance time, and swing time) calculated using gait events extracted from our recently developed machine learning model [10]. We hypothesized that all parameters would present low rRMSE (<5%) and a moderate-to-strong association between experimental and predicted parameters (Pearson’s correlation coefficient > 0.6).

## 2. Materials and Methods

### 2.1. Participants

Thirty-five males (age: 33.0 ± 13.0 years, height: 181.4 ± 7.1 cm, body mass: 88.1 ± 15.2 kg) and seventeen females (age: 34.3 ± 13.7 years, height: 169.4 ± 6.3 cm, body mass: 72.2 ± 8.9 kg) participated in the study. None had prior neurological or musculoskeletal injuries that would prevent treadmill walking. Participants were verbally informed about the experimental procedure and provided both verbal as well as written informed consent before participation. All methods were conducted in accordance with relevant guidelines and regulations from the Declaration of Helsinki (2004), and the experimental procedure was approved by the local Research Ethics Committee (Case number: 2023-505-00041).

### 2.2. Experimental Design and Instrumentation

The experimental protocol has been described in detail elsewhere [10]. Briefly, participants walked for between 80 and 110 s on a motorized treadmill at three different speeds (1 m/s, 1.25 m/s, and 1.5 m/s) while carrying a 3D-printed surrogate smartphone case containing an IMU. Participants carried surrogate smartphones in the right pocket of their jacket and trousers or in their right hand. The surrogate smartphone (dimensions: 15 × 8.5 × 1.1 cm; weight: 0.167 Kg, including a regular smartphone battery) contained a slot to fit an IMU (Trigno Avanti Sensor, Delsys Inc., Natick, MA, USA). We acquired three-axial acceleration (ACC, ±16 g) and angular rate data (AR, ±2000°/s) at 1259 Hz. Additionally, we captured the 3D positions of retro-reflective markers using a 12-camera optical motion capture system (QTM 2023.2, Qualisys, Göteborg, Sweden) at 100 Hz. Markers were placed on the participant’s walking shoes to represent their calcaneus and first metatarsal bilaterally, to extract heel strike and toe-off events. The marker and IMU data were recorded through the motion capture software (QTM 2023.2, Qualisys, Göteborg, Sweden).

### 2.3. Data Processing

The raw IMU data were downsampled to 100 Hz to match the sampling rate of the optical marker system. Both the IMU signals and the optical marker data were low-pass filtered using a 4th-order Butterworth filter with a cutoff frequency of 10 Hz to remove high-frequency noise and motion artifacts. Heel strike and toe-off events were identified using a proprietary event detection algorithm based on the 3D positions of retro-reflective markers placed on the participants’ shoes. Specifically, heel strike was defined as the local minimum in the vertical position of the calcaneus marker within the first 30% of the interval between successive foot motion peaks. Toe-off was identified as the local minimum of the metatarsal marker occurring between 40% and 80% of that same interval. These gait events served as ground-truth labels for subsequent IMU-based analysis. The synchronized IMU data were segmented into 200 ms windows with a 100 ms overlap. A window was labeled as containing a gait event only if the event occurred near the center of the window (between the 45th and 55th percentile of the window length), ensuring accurate temporal localization. All IMU data were normalized using a Robust Scaler to reduce the influence of outliers. We used a machine learning pipeline to predict gait events from the IMU data. The pipeline consisted of a convolutional neural network followed by long short-term memory (LSTM) networks and was trained using an 80/20 train–test split. We used the results from this leave-one-out approach to calculate clinically relevant gait parameters. The stride time was defined as the time between two sequential heel strikes. The stance time was defined as the time from a heel strike to the subsequent ipsilateral toe-off. The swing time was defined as the time from a toe-off to the subsequent ipsilateral heel strike. The double-support time was defined as the time in which both feet were simultaneously in stance phase. Initially, 28,797 stride cycles from 43 participants were included in the analysis (80 ± 5 stride cycles from each participant in each IMU sensor). A subsequent quality assessment excluded 5234 erroneous gait cycles, primarily due to condition transitions or implausible negative time durations. After filtering, an average of 67 ± 23 valid stride cycles remained for each condition and walking speed combination. No differences between left and right sides were detected in the previous analysis. Therefore, the data from left and right sides were combined during the analysis.

### 2.4. Statistical Analysis

To evaluate the accuracy of gait parameter predictions derived from smartphone position data, we conducted several statistical analyses. Both experimental (M1) and predicted (M2) gait parameters—double support time, stride time, stance time, and swing time—were visualized using violin plots to represent data distribution and variability. Prediction accuracy was quantified using the relative root mean square error (rRMSE), calculated between M1 and M2 for each smartphone location (hand, trouser pocket, and jacket pocket) and walking speed condition (1.00 m/s, 1.25 m/s, and 1.50 m/s). The inter-subject association between the experimental and predicted values was assessed using Pearson correlation analyses. The strength of the correlation was interpreted using standard thresholds: weak (r < 0.3), moderate (0.3 ≤ r < 0.6), and strong (r ≥ 0.6) [11]. Additionally, Bland–Altman plots were used to assess agreement between M1 and M2. The Bland–Altman analysis included all subjects and measurements across all conditions. Each data point represented a single gait parameter estimate for a subject at a specific speed and sensor location. The mean difference (bias) and 95% limits of agreement were calculated to evaluate systematic bias and random error between M1 and M2.

## 3. Results

### 3.1. Comparison Experimental vs. Predicted Gait Parameters

**Figure 1:**
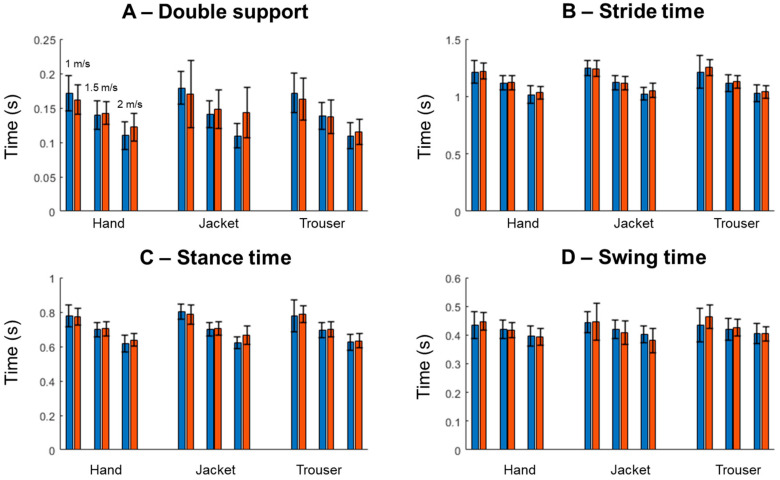
The mean ± standard deviation of the experimental (blue) and predicted (red) double support (**A**), stride time (**B**), stance time (**C**), and swing time (**D**) when participants walk with the mock smartphone in their hand, jacket pocket, or trouser pocket. For each condition, the three pairs of bars represent walking speeds of 1.0 m/s, 1.25 m/s, and 1.5 m/s, respectively.

The violin plots in Figure 1 illustrate the overall distribution of the double support (A), stride time (B), stance time (C), and swing time (D). All parameters demonstrate similar distributions between the experimental and predicted gait parameters, except double support from the jacket location (Figure 1A). Additionally, outliers were observed in the predicted double support and swing time from the jacket location. Experimental stride time and stance time from the hand and trouser locations also showed outliers.

### 3.2. Relative Error in Predicting Gait Parameters

The rRMSE for predicting the double support time revealed errors exceeding 25%, reaching up to 75% (Table 1). Predictions from the jacket location showed the highest errors across all walking speeds. Conversely, the rRMSE for predicting stride time remains predominantly below 5%. The fastest speed (1.5 m/s) shows the highest rRMSE, particularly for predictions from the jacket location. For stance time, the rRMSE ranges between 5% and 15%, again with the jacket location yielding the highest errors across all speeds. Similarly, the rRMSE for predicting swing time from the jacket reaches the highest values (between 17% and 21%), especially at 1.5 m/s. The distribution of the rRMSE within each combination of walking speed and sensor placement was usually skewed (skewness mean (SD): 2.5 (1.2); minimum: 0; 75, maximum: 5.3). Due to such skewness, some of the median values in Table 1 are below the lower-bound of the 95% confidence interval. Symmetry between the right and left stride and stance times is also evaluated (Supplementary Table S1). Stride time symmetry demonstrated low rRMSE values of between 3% and 10%. In contrast, stance time symmetry showed higher variability, with errors ranging from 10% to 30%. For both parameters, the lowest errors were observed at the fastest walking speed (1.5 m/s).

### 3.3. Inter-Subject Association Between the Experimental and Predicted Gait Parameters

**Figure 2:**
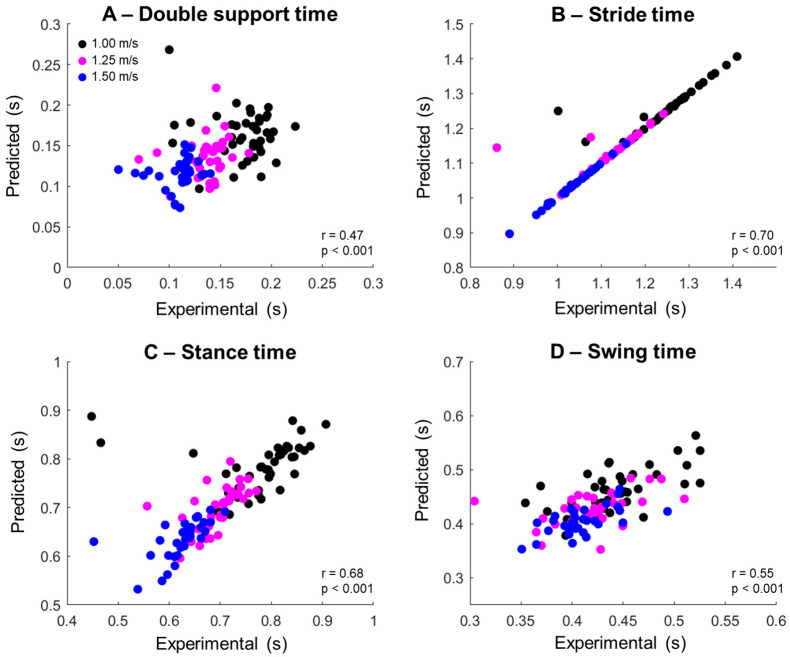
Linear regressions of the association between experimental and predicted double support time (**A**), stride time (**B**), stance time (**C**), and swing time (**D**) extracted from the trouser position at 1.0 m/s (black dots), 1.25 m/s (cyan dots), and 1.5 m/s (blue dots). Pearson’s correlation coefficient (r) and the significance level (p) were computed for each combined association.

Illustrative regressions from the measurements extracted from the trouser position, combining all walking speeds, are shown in Figure 2. In general, there are significant moderate associations between the experimental and predicted variables. Specifically, there are no significant associations for double support, whereas all other parameters show moderate to strong associations (Table 2). Predictions for stride time show the highest associations and were predominantly above 0.7.

**Figure 3:**
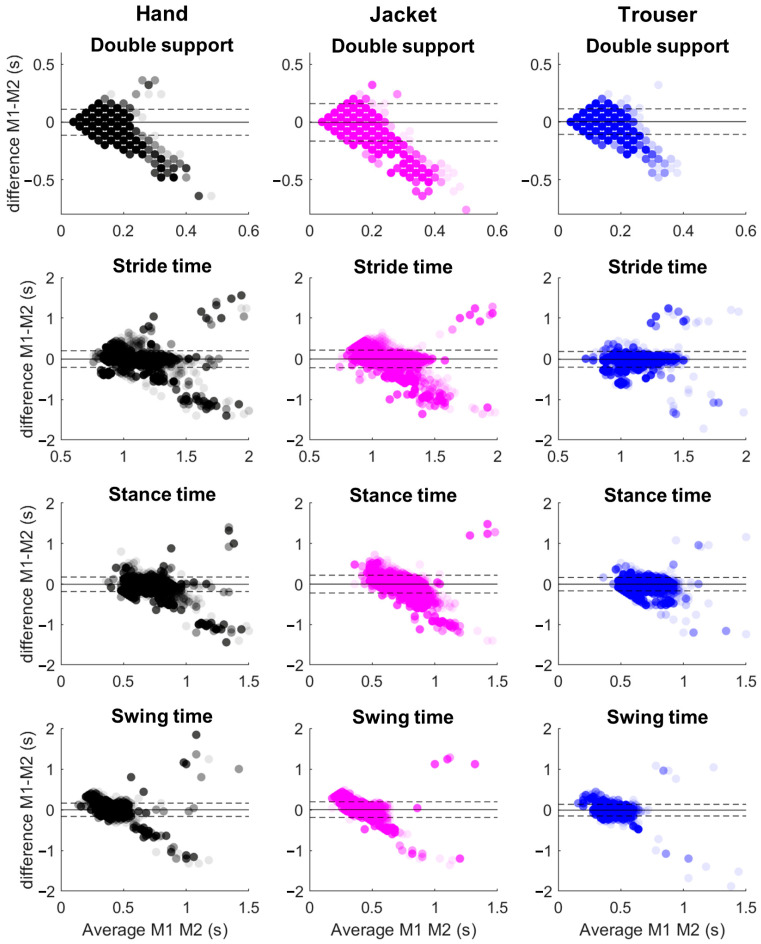
Bland–Altman plots comparing experimental (M1) and predicted (M2) gait parameters—double support (top row), stride time (second row), stance time (third row), and swing time (bottom row)—extracted from smartphone data located at the hand (left column, black), jacket (middle column, magenta), and trouser pocket (right column, blue). Each dot represents a single measurement from one subject and walking speed. The y-axis shows the difference between M1 and M2 (M1–M2), while the x-axis shows their average. The solid horizontal line indicates the mean bias, while the dashed lines indicate the 95% limits of agreement (±1.96 SD from the mean difference).

The Bland–Altman plots (Figure 3) show that the mean bias across all sensor locations and walking speeds ranged from −0.032 s to 0.02 s. The majority of predictions (>95%) fell within the limits of agreement, although some data points lay outside these bounds.

## 4. Discussion

The main finding of this study was that the stride time predictions generally exhibited relative root mean square errors (rRMSEs) below 5%, while double support times showed higher errors, typically above 20%. Accuracy was highest when the surrogate smartphone was positioned on the trouser or hand. Additionally, moderate-to-strong correlations were observed between the experimental and predicted stride, stance, and swing times. These associations were consistent across sensor locations and walking speeds, highlighting the model’s sensitivity to inter-subject variability. Since these spatiotemporal parameters are directly computed from the estimated timing of gait events, the quality of event detection critically determines the accuracy of the derived metrics. Our results indicate that the model’s ability to identify gait events translates into clinically meaningful estimates of stride-related parameters. Thus, the proposed machine learning method shows promise not only for gait event detection but also for continuous gait monitoring in real-world conditions, where the accurate tracking of timing-based gait features is essential for clinical assessment.

### 4.1. Parameter-Dependent Relative Errors

The double support time yields the highest relative error time (range: 29.9–73.4%), whereas the stride time presented the lowest error (range: 2.4–6.0%). One explanation for the error disparity is the relative magnitude of the parameters. Double support durations are short (0.1–0.2 s), while stride times are longer (1–1.5 s), which affects how prediction errors scale. A limitation of the applied machine learning algorithm is the need for fixed time windows—such windows may vary by a few milliseconds to a few seconds [12]. Therefore, the longer the window, the greater the error that a single window represents. Our time windows were 200 ms long. A misalignment of just one window leads to a 200 ms error, which significantly affects the prediction quality of short-duration parameters like double support time. Conversely, the stride time presented the longest duration and was the only parameter identified from a single type of gait event (e.g., the heel strike event for a given side), possibly optimizing its prediction quality. Each gait event has been predicted using independently trained algorithms, potentially increasing the variability of double support time, stance, and swing times. Previously, Liu et al. found a rRMSE when predicting stride times of approximately 15% [9] by applying older peak detection algorithms. Our results, particularly for stride time extraction, show superior prediction quality compared to previous studies. This is notable given the lack of IMU fixation, the use of multiple sensor locations, and varying walking speeds. Moreover, the inferior quality in predicting gait parameters using the data from the jacket pocket aligns with our previous findings of inferior quality in predicting heel strikes and toe-offs from this sensor location [10], although the magnitude of the differences were substantially lower for gait events. It is plausible that walking with the sensor in the jacket pocket increases random motion artifacts [10]. Seo et al. [3] reported greater errors from predicting gait events from an IMU placed in the trouser pocket (MAE ≈ 0.06 s) compared to placing it on the pelvis (MAE ≈ 0.03 s), corroborating our assumption that the lack of sensor fixation will negatively influence prediction quality.

### 4.2. Fixed vs. Non-Fixed IMU Placement for Gait Assessments

The use of a single IMU to predict spatiotemporal gait parameters has been explored predominantly in laboratory settings. Pepa et al. reported excellent associations (r > 0.89) and no systematic bias when predicting step times using a peak detection algorithm applied to IMU data from a sensor fixed to the lower back [1]. Liu et al. reported excellent associations (r > 0.99) in predicting stride times from an IMU sensor fixed to the wrist of a small homogenous sample (*n* = 8) [9]. Laboratory walking data yields low inter-trial variability and optimally detectable peaks due to the tightly fixed IMUs. However, the IMU data collected from loosely fitted locations, as explored in this study, may limit the performance of peak detection-based algorithms. This setup hinders the accurate identification of gait events.

A study using data from an IMU fixed to the shank and machine learning algorithms showed no systematic time error bias when predicting gait events in both healthy and neurological cohorts [2]. Placing IMUs on the shank and foot yields the optimal conditions for predicting spatiotemporal gait parameters using machine learning, whereas the predictions are less accurate when placing sensors on the thigh or wrist [3]. Smartphone apps such as OneStep apply machine learning to analyze smartphone IMU data when the device is used in the front trouser pocket [4,5,6,8]. Several studies have used this app to perform gait analysis, but there are substantial inconsistencies regarding how smartphones are fixed and the ground-truth methods. Shema-Shiratzky reported strong correlations and low systematic bias when comparing predicted stride time from the app to gait cycles measured with a pressure mat, whereas predictions of stance time were only moderately correlated to the values extracted from the pressure mat (r < 0.55) [4]. However, the authors fixed the phone to the thigh instead of measuring from the trouser pocket. Christensen et al. and Contreras et al. validated estimations of swing and stance time (ICC range: 0.72–0.97) from the OneStep measurements against optical motion capture [5,6], while Shahar and Agmon validated the OneStep measurements for swing and stance time (ICC range: 0.83–0.90) against another method using IMU data [8]. Both Shema-Shiratzky et al. and Contreras et al. found moderate associations between the swing times extracted from OneStep and their claimed gold standards (r = 0.46–0.73) [4,6], and all studies presented systematic error bias between predicted and measured swing times using the OneStep algorithm [4,5,6,8]. Interestingly, Shema-Shiratzky et al. and Contreras et al. estimated double support times, which were more stable compared to the method presented in our study (r = 0.61–0.80) [4,6], but double support predictions also presented a systematic time error bias between predictions and the claimed gold standards [4,5,6,8]. In general, our results represent similar or superior performance compared to the results from similar techniques. The use of CNN-LSTM algorithms to estimate gait parameters represents a substantial advance in gait biomechanics. Notably, gait variable extraction was unaffected by sensor location, walking speed, or how tightly clothes fit the sensor. Future studies should focus on estimating gait parameters using high-quality methods to establish the ground truth, preferably ground reaction forces or, alternatively, optical motion capture.

The observed variability in rRMSE between different gait parameters, particularly for double support time, may limit clinical utility. Clinicians typically place more trust in camera-based systems than in wearable technologies [13]. Interestingly, clinicians assess only the average gait data over a period of time [14,15], such as the average stride time within 2 or 5 min. Our proposed method is relevant in the context of data averaging, since no systematic bias was found between the experimental and predicted gait parameters (see Figure 3), demonstrating that individuals with longer stride times or swing times within a sample are identifiable from our predictions. Gait event detection algorithms are usually based on peak detection in time-series data [16]. In contrast to machine learning algorithms that usually need sliding windows, peak detection methods can precisely identify peaks/valleys. However, peak-detection-based algorithms do not always align perfectly with relevant gait events and are susceptible to noise or motion artifacts [17,18], demanding the creation of more robust methods to identify gait events. We previously demonstrated that deep learning algorithms can detect such gait events from a wide range of conditions, such as sensor orientation, loose clothes artifacts, or multiple phone positions [10], which is a necessity for real-world applications.

### 4.3. Limitations of the Study

Firstly, our results are based on a sample of young adults. Contreras et al. also demonstrated variance in prediction accuracy across diverse types of pathological gait disorders [6]. Therefore, future studies evaluating machine learning algorithms should include gait data from older adults and people with gait disorders. Secondly, our walking task was performed on a regular treadmill for a quicker and easier assessment of heel strikes and toe-offs using optical motion capture. However, the gold-standard method for detecting gait events involves recording ground reaction forces. This limitation hinders the true accuracy of our gait parameter estimation; however, studies comparing the estimation of gait events from optical motion capture to ground reaction forces revealed errors within 15 ms [19,20], whereas greater errors are found from estimations based on single IMUs (>50 ms, up to 250 ms) [19]. Finally, treadmill walking hinders capturing natural gait variability, since treadmill walking may decrease stride time, swing time, and stance time, while prolonging double support time [21]. Therefore, future studies should aim at evaluating our method using overground walking, preferably in outdoor conditions to increase the ecological validity of the dataset.

## 5. Conclusions

The present study demonstrated that predictions of walking stride time presented low relative errors (rRMSE < 5%), while stance and swing times exhibited low-to-moderate errors (rRMSE between 6 and 20%). However, predictions of double support time presented substantial relative errors (rRMSE > 20%), which might be caused by the long time-window selected for the event detection deep learning algorithm. Despite these variations, there were moderate-to-strong associations between the predicted and experimental gait parameters, suggesting the potential of the developed method to track gait quality in natural settings regardless of sensor placement or walking speed.

## Figures and Tables

**Table 1:** Relative root mean square error (rRMSE, in %) for double support time, stride time, stance time, and swing time across the different walking speeds and sensor placements.

		Double Support Time				Stride Time			
		Median	IQR	CI Low	CI High	Median	IQR	CI Low	CI High
	**Hand**	31.1	21.0	30.7	40.5	3.6	3.0	3.6	8.0
**1.00**	**Jacket**	42.7	20.8	40.3	54.2	4.5	4.7	4.6	8.5
	**Trouser**	35.0	19.0	30.6	50.0	2.6	1.4	1.9	13.6
	**Hand**	29.9	22.5	30.9	42.3	3.0	1.6	3.2	5.3
**1.25**	**Jacket**	46.9	27.8	45.1	60.5	4.6	6.1	5.2	9.7
	**Trouser**	32.7	20.7	31.1	44.3	2.4	0.9	1.8	5.4
	**Hand**	47.4	21.2	43.4	60.4	3.3	5.1	3.8	9.7
**1.50**	**Jacket**	73.4	72.3	71.8	109.7	6.0	11.4	6.4	14.2
	**Trouser**	34.4	23.7	33.8	52.3	2.4	0.6	1.4	5.8
		**Stance Time**				**Swing Time**			
		**Median**	**IQR**	**CI Low**	**CI High**	**Median**	**IQR**	**CI Low**	**CI High**
	**Hand**	7.5	4.6	7.5	11.3	13.2	8.8	13.3	19.7
**1.00**	**Jacket**	10.3	5.4	10.4	14.8	17.7	11.1	18.6	27.1
	**Trouser**	7.6	4.1	6.5	18.8	13.4	8.1	12.8	23.1
	**Hand**	7.2	5.1	7.1	10.0	11.5	7.3	11.3	14.5
**1.25**	**Jacket**	10.1	7.3	9.6	15.6	17.0	10.0	16.0	22.5
	**Trouser**	7.3	4.2	6.6	9.7	11.3	6.1	10.2	15.4
	**Hand**	10.6	9.6	9.6	16.1	13.5	7.2	12.6	18.0
**1.5**	**Jacket**	15.7	19.0	15.9	26.2	20.7	19.8	19.8	29.1
	**Trouser**	6.2	3.4	5.7	10.4	9.9	4.9	9.3	13.5

**Table 2:** Pearson correlation coefficients (r) from the association between experimental and predicted double support, stride time, stance time, and swing time. * Denotes *p* < 0.05. ** Denotes *p* < 0.001.

		Double Support	Stride Time	Stance Time	Swing Time
	**Hand**	−0.001	0.804 **	0.695 **	0.492 *
**1.0 m/s**	**Jacket**	−0.151	0.989 **	0.761 **	0.535 **
	**Trouser**	0.026	0.614 *	0.438 *	0.451 *
	**Hand**	0.060	0.951 **	0.527 **	0.484 **
**1.25 m/s**	**Jacket**	−0.092	0.941 **	0.480 *	0.525 **
	**Trouser**	0.255	0.554 *	0.498 *	0.296
	**Hand**	−0.130	0.344 *	0.086 *	0.541 **
**1.5 m/s**	**Jacket**	0.241	0.727 **	0.608 **	0.464 *
	**Trouser**	0.160	0.744 **	0.614 **	0.545 **

## Data Availability

Data will be available upon request to the authors.

## Supplementary Materials

The following supporting information can be downloaded at: https://www.mdpi.com/article/10.3390/s25144470/s1, Table S1: Relative root mean square error (rRMSE, in %) from the left/right symmetry of stride time and stance time.
