# Supplementary material for: Extraction of Clinically Relevant Temporal Gait Parameters from IMU Sensors Mimicking the Use of Smartphones
Source: Sensors (Basel). 2025 Jul 18;25(14):4470. doi: 10.3390/s25144470 (PMC12299727; doi:10.3390/s25144470)
Supplement: Supplementary file 1 [file sensors-25-04470-s001.zip › sensors-3683247-supplementary.pdf]

Supplementary Table S1. Relative root mean square error (rRMSE, in %) from the left/right symmetry of stride time and stance time.

		symmetry stride time				symmetry stance time			
		**Me-dian**	**IQR**	**CI low**	**CI high**	**Median**	**IQR**	**CI low**	**CI high**
**1**	**Hand**	4.4	4.1	5.2	9.1	9.7	4.3	6.1	27.8
**1**	**Jacket**	5.9	6.6	6.3	10.5	15.3	15.5	15.8	32.9
**1**	**Trouser**	3.6	1.6	3.5	4.8	9.6	5.8	8.3	15.3
**1.25**	**Hand**	4.2	3.1	4.3	5.8	8.9	7.1	9.1	12.3
**1.25**	**Jacket**	5.7	7.3	6.8	12.4	14.7	9.7	13.1	29.2
**1.25**	**Trouser**	3.3	1.4	3.2	4.1	9.0	5.8	8.4	11.6
**1.5**	**Hand**	4.1	4.1	4.6	6.5	12.7	8.5	11.1	14.5
**1.5**	**Jacket**	6.4	8.7	7.0	13.6	21.1	16.3	20.3	35.4
**1.5**	**Trouser**	3.3	0.7	3.0	4.0	9.3	3.6	8.4	11.4
